# 6-Benzyl-6,7-dihydro-5*H*-pyrrolo­[3,4-*b*]pyridine-5,7-dione

**DOI:** 10.1107/S1600536811041006

**Published:** 2011-10-08

**Authors:** Hong-Shun Sun, Long Jiang, Hong Xu, Xin-Hua Lu, Yu-Long Li

**Affiliations:** a Department of Applied Chemistry, Nanjing College of Chemical Technology, Nanjing 210048, People’s Republic of China; b R&D Center, Jiangsu Yabang Pharmaceutical Group, Changzhou 213200, People’s Republic of China; c Department of Chemical Engineering, Nanjing College of Chemical Technology, Nanjing 210048, People’s Republic of China

## Abstract

In the title compound, C_14_H_10_N_2_O_2_, the dihedral angle between the heterocyclic ring system and the phenyl ring is 45.8 (5)°. Weak inter­molecular C—H⋯N hydrogen bonding is present in the crystal structure.

## Related literature

The title compound is a key inter­mediate in the synthesis of the quinolone anti­biotic moxifloxacin [systematic name: 1-cyclo­propyl-7-[(1*S*,6*S*)-2,8-diaza­bicyclo­[4.3.0]non-8-yl]-6-fluoro-8-meth­oxy-4-oxo-quinoline-3-carb­oxy­lic acid], see: Petersen *et al.* (1993 ▶). For a related structure, see: Garduño-Beltrán *et al.* (2009 ▶).
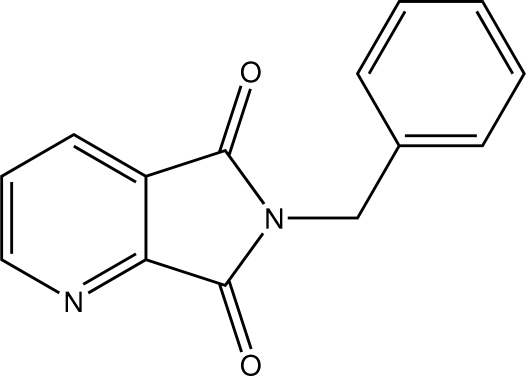


## Experimental

### 

#### Crystal data

C_14_H_10_N_2_O_2_
*M* *_r_* = 238.24
Monoclinic, 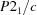

*a* = 11.8548 (6) Å
*b* = 12.3969 (8) Å
*c* = 8.1676 (4) Å
β = 107.45 (3)°
*V* = 1145.1 (2) Å^3^
*Z* = 4
Mo *K*α radiation
μ = 0.10 mm^−1^
*T* = 293 K
0.20 × 0.10 × 0.10 mm

#### Data collection

Enraf–Nonius CAD-4 diffractometer
Absorption correction: ψ scan (North *et al.*, 1968 ▶) *T*_min_ = 0.981, *T*_max_ = 0.991
2087 measured reflections
2087 independent reflections
1100 reflections with *I* > 2σ(*I*)
*R*_int_ = 0.045
3 standard reflections every 200 reflections  intensity decay: 1%

#### Refinement

*R*[*F* ^2^ > 2σ(*F* ^2^)] = 0.058
*wR*(*F* ^2^) = 0.174
*S* = 1.00
2087 reflections
163 parameters
12 restraints
H-atom parameters constrained
Δρ_max_ = 0.17 e Å^−3^
Δρ_min_ = −0.13 e Å^−3^

### 

Data collection: *CAD-4 EXPRESS* (Enraf–Nonius, 1994 ▶); cell refinement: *CAD-4 EXPRESS*; data reduction: *XCAD4* (Harms & Wocadlo, 1995 ▶); program(s) used to solve structure: *SHELXTL* (Sheldrick, 2008 ▶); program(s) used to refine structure: *SHELXTL*; molecular graphics: *SHELXTL*; software used to prepare material for publication: *SHELXTL*.

## Supplementary Material

Crystal structure: contains datablock(s) I, global. DOI: 10.1107/S1600536811041006/xu5342sup1.cif

Structure factors: contains datablock(s) I. DOI: 10.1107/S1600536811041006/xu5342Isup2.hkl

Supplementary material file. DOI: 10.1107/S1600536811041006/xu5342Isup3.cml

Additional supplementary materials:  crystallographic information; 3D view; checkCIF report

## Figures and Tables

**Table 1:** Hydrogen-bond geometry (Å, °)

*D*—H⋯*A*	*D*—H	H⋯*A*	*D*⋯*A*	*D*—H⋯*A*
C10—H10*A*⋯N2^i^	0.93	2.46	3.386 (3)	177

Symmetry code: (i) 
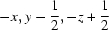
.

**Fig. 1.**
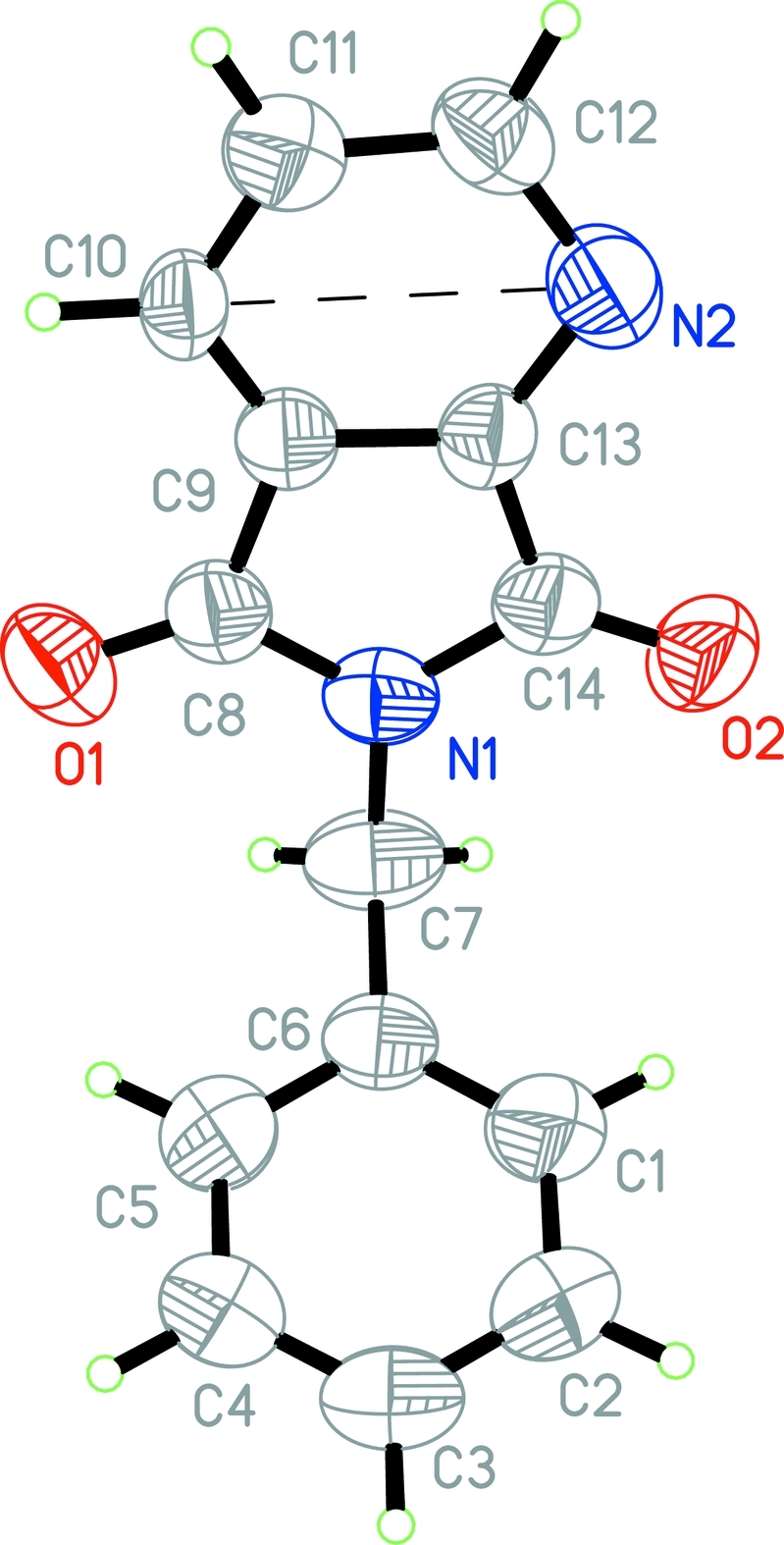
The molecular structure of (I), showing the atom-numbering scheme and displacement ellipsoids at the 50% probability level.

## supplementary crystallographic information

### Comment

Moxifloxacin (Petersen *et al.*, 1993) is used to treat a variety of bacterial infections. This medication belongs to a class of drugs called quinolone antibiotics. The title compound is a key intermediate to synthesize it. As part of our studies in this area, we report here its crystal structure.

In the title compound, all bond lengths and angles show normal values. The dihedral angle between the heterocycle and benzyl group is 45.8 (5)° (Fig.1), similar to that found in a related strcture (Garduño-Beltrán *et al.*, 2009). There is a intermolecular C—H···N hydrogen bond (Table 1) in the crystal structure.

### Experimental

Benzylamine (3.85 ml, 35.2 mmol) was added to a suspension of 2,3-pyridinedicarboxylic anhydride (5 g, 33.5 mmol) in acetic acid (50 ml), and the mixture was heated under reflux for 18 h. It was then cooled to room temperature, concentrated *in vacuo* and the residue was triturated with diethyl ether to afford the title compound as a white solid in 57% yield. Crystals of (I) suitable for X-ray diffraction were obtained by slow evaporation of an acetone solution.

### Refinement

All H atoms were positioned geometrically and refined using a riding model with C—H = 0.93–0.97 Å and *U*_iso_(H) = 1.2 *U*_eq_(C).

### Crystal data

**Table:**

C_14_H_10_N_2_O_2_	*F*(000) = 496
*M**_r_* = 238.24	*D*_x_ = 1.382 Mg m^−^^3^
Monoclinic, *P*2_1_/*c*	Mo *K*α radiation, λ = 0.71073 Å
Hall symbol: -P 2ybc	Cell parameters from 25 reflections
*a* = 11.8548 (6) Å	θ = 10–13°
*b* = 12.3969 (8) Å	µ = 0.10 mm^−^^1^
*c* = 8.1676 (4) Å	*T* = 293 K
β = 107.45 (3)°	Block, colorless
*V* = 1145.1 (2) Å^3^	0.20 × 0.10 × 0.10 mm
*Z* = 4	

### Data collection

**Table:**

Enraf–Nonius CAD-4 diffractometer	1100 reflections with *I* > 2σ(*I*)
Radiation source: fine-focus sealed tube	*R*_int_ = 0.045
graphite	θ_max_ = 25.4°, θ_min_ = 1.8°
ω/2θ scans	*h* = −14→13
Absorption correction: ψ scan (North *et al.*, 1968)	*k* = 0→14
*T*_min_ = 0.981, *T*_max_ = 0.991	*l* = 0→9
2087 measured reflections	3 standard reflections every 200 reflections
2087 independent reflections	intensity decay: 1%

### Refinement

**Table:**

Refinement on *F*^2^	Primary atom site location: structure-invariant direct methods
Least-squares matrix: full	Secondary atom site location: difference Fourier map
*R*[*F*^2^ > 2σ(*F*^2^)] = 0.058	Hydrogen site location: inferred from neighbouring sites
*wR*(*F*^2^) = 0.174	H-atom parameters constrained
*S* = 1.00	*w* = 1/[σ^2^(*F*_o_^2^) + (0.082*P*)^2^] where *P* = (*F*_o_^2^ + 2*F*_c_^2^)/3
2087 reflections	(Δ/σ)_max_ < 0.001
163 parameters	Δρ_max_ = 0.17 e Å^−^^3^
12 restraints	Δρ_min_ = −0.13 e Å^−^^3^

### Special details

**Table:**

Geometry. All e.s.d.'s (except the e.s.d. in the dihedral angle between two l.s. planes) are estimated using the full covariance matrix. The cell e.s.d.'s are taken into account individually in the estimation of e.s.d.'s in distances, angles and torsion angles; correlations between e.s.d.'s in cell parameters are only used when they are defined by crystal symmetry. An approximate (isotropic) treatment of cell e.s.d.'s is used for estimating e.s.d.'s involving l.s. planes.
Refinement. Refinement of *F*^2^ against ALL reflections. The weighted *R*-factor *wR* and goodness of fit *S* are based on *F*^2^, conventional *R*-factors *R* are based on *F*, with *F* set to zero for negative *F*^2^. The threshold expression of *F*^2^ > σ(*F*^2^) is used only for calculating *R*-factors(gt) *etc*. and is not relevant to the choice of reflections for refinement. *R*-factors based on *F*^2^ are statistically about twice as large as those based on *F*, and *R*- factors based on ALL data will be even larger.

### Fractional atomic coordinates and isotropic or equivalent isotropic displacement parameters (Å^2^)

**Table:**

	*x*	*y*	*z*	*U*_iso_*/*U*_eq_	
N1	0.2267 (2)	0.6223 (2)	0.0923 (3)	0.0602 (7)	
O1	0.20765 (18)	0.43903 (17)	0.1329 (3)	0.0753 (7)	
C1	0.4977 (3)	0.7166 (3)	0.2368 (5)	0.0896 (11)	
H1A	0.4640	0.7810	0.1871	0.108*	
N2	−0.0141 (2)	0.74370 (19)	0.2194 (3)	0.0711 (7)	
O2	0.19962 (18)	0.80705 (17)	0.0885 (3)	0.0782 (7)	
C2	0.6005 (3)	0.7199 (3)	0.3733 (5)	0.0957 (12)	
H2B	0.6327	0.7861	0.4170	0.115*	
C3	0.6539 (3)	0.6287 (3)	0.4429 (4)	0.0763 (9)	
H3A	0.7242	0.6307	0.5324	0.092*	
C4	0.6038 (3)	0.5337 (3)	0.3807 (5)	0.0980 (13)	
H4A	0.6398	0.4698	0.4289	0.118*	
C5	0.5005 (3)	0.5297 (3)	0.2473 (5)	0.0919 (12)	
H5A	0.4681	0.4630	0.2066	0.110*	
C6	0.4446 (2)	0.6219 (2)	0.1734 (3)	0.0577 (8)	
C7	0.3308 (3)	0.6186 (3)	0.0307 (4)	0.0758 (10)	
H7A	0.3282	0.6793	−0.0454	0.091*	
H7B	0.3282	0.5530	−0.0351	0.091*	
C8	0.1751 (2)	0.5306 (2)	0.1379 (3)	0.0570 (8)	
C9	0.0735 (2)	0.5705 (2)	0.1955 (3)	0.0505 (6)	
C10	−0.0010 (2)	0.5161 (2)	0.2533 (3)	0.0493 (7)	
H10A	0.0040	0.4415	0.2653	0.059*	
C11	−0.0833 (3)	0.5716 (2)	0.2939 (4)	0.0655 (8)	
H11A	−0.1372	0.5342	0.3353	0.079*	
C12	−0.0940 (3)	0.6819 (2)	0.2785 (3)	0.0633 (8)	
H12A	−0.1550	0.7164	0.3075	0.076*	
C13	0.0710 (2)	0.6813 (2)	0.1768 (3)	0.0504 (6)	
C14	0.1696 (3)	0.7163 (2)	0.1142 (3)	0.0581 (8)	

### Atomic displacement parameters (Å^2^)

**Table:**

	*U*^11^	*U*^22^	*U*^33^	*U*^12^	*U*^13^	*U*^23^
N1	0.0490 (14)	0.0711 (16)	0.0568 (14)	0.0019 (13)	0.0100 (11)	0.0026 (13)
O1	0.0755 (15)	0.0661 (14)	0.0803 (15)	0.0207 (12)	0.0173 (12)	−0.0042 (11)
C1	0.063 (2)	0.075 (2)	0.118 (3)	−0.0018 (18)	0.007 (2)	0.021 (2)
N2	0.0711 (18)	0.0628 (14)	0.0755 (17)	0.0089 (11)	0.0162 (13)	−0.0014 (12)
O2	0.0702 (15)	0.0627 (13)	0.0928 (16)	−0.0110 (11)	0.0108 (12)	0.0161 (11)
C2	0.067 (2)	0.084 (3)	0.120 (3)	−0.014 (2)	0.003 (2)	−0.005 (2)
C3	0.0509 (18)	0.101 (3)	0.076 (2)	−0.001 (2)	0.0185 (16)	0.001 (2)
C4	0.068 (2)	0.079 (3)	0.129 (3)	0.009 (2)	0.003 (2)	0.014 (2)
C5	0.073 (3)	0.072 (2)	0.113 (3)	−0.0044 (19)	0.000 (2)	−0.010 (2)
C6	0.0490 (16)	0.071 (2)	0.0572 (16)	0.0014 (16)	0.0227 (14)	0.0005 (16)
C7	0.0562 (19)	0.114 (3)	0.0586 (18)	−0.0033 (18)	0.0195 (16)	0.0010 (18)
C8	0.0532 (18)	0.0573 (19)	0.0503 (17)	0.0024 (15)	−0.0002 (13)	−0.0016 (13)
C9	0.0483 (15)	0.0511 (12)	0.0446 (15)	0.0002 (12)	0.0027 (12)	−0.0005 (12)
C10	0.0542 (17)	0.0359 (12)	0.0503 (15)	0.0001 (10)	0.0040 (12)	0.0023 (11)
C11	0.0599 (18)	0.0689 (14)	0.0665 (19)	0.0027 (14)	0.0171 (15)	0.0000 (16)
C12	0.0601 (19)	0.0673 (15)	0.0618 (18)	0.0126 (14)	0.0171 (14)	−0.0092 (16)
C13	0.0525 (15)	0.0481 (12)	0.0442 (14)	−0.0032 (12)	0.0049 (12)	0.0002 (12)
C14	0.0516 (18)	0.060 (2)	0.0516 (17)	−0.0023 (15)	−0.0014 (13)	0.0026 (14)

### Geometric parameters (Å, °)

**Table:**

N1—C14	1.385 (4)	C4—H4A	0.9300
N1—C8	1.394 (3)	C5—C6	1.367 (4)
N1—C7	1.467 (4)	C5—H5A	0.9300
O1—C8	1.203 (3)	C6—C7	1.496 (4)
C1—C6	1.359 (4)	C7—H7A	0.9700
C1—C2	1.384 (5)	C7—H7B	0.9700
C1—H1A	0.9300	C8—C9	1.503 (4)
N2—C13	1.395 (3)	C9—C10	1.306 (3)
N2—C12	1.411 (4)	C9—C13	1.382 (4)
O2—C14	1.217 (3)	C10—C11	1.315 (4)
C2—C3	1.336 (4)	C10—H10A	0.9300
C2—H2B	0.9300	C11—C12	1.376 (4)
C3—C4	1.347 (4)	C11—H11A	0.9300
C3—H3A	0.9300	C12—H12A	0.9300
C4—C5	1.374 (4)	C13—C14	1.474 (4)
			
C14—N1—C8	112.4 (2)	N1—C7—H7B	109.0
C14—N1—C7	124.4 (3)	C6—C7—H7B	109.0
C8—N1—C7	123.2 (3)	H7A—C7—H7B	107.8
C6—C1—C2	121.9 (3)	O1—C8—N1	126.2 (3)
C6—C1—H1A	119.1	O1—C8—C9	128.0 (3)
C2—C1—H1A	119.1	N1—C8—C9	105.8 (2)
C13—N2—C12	113.2 (2)	C10—C9—C13	124.0 (3)
C3—C2—C1	120.5 (3)	C10—C9—C8	129.5 (3)
C3—C2—H2B	119.7	C13—C9—C8	106.5 (3)
C1—C2—H2B	119.7	C9—C10—C11	117.1 (3)
C2—C3—C4	118.7 (3)	C9—C10—H10A	121.5
C2—C3—H3A	120.7	C11—C10—H10A	121.5
C4—C3—H3A	120.7	C10—C11—C12	123.4 (3)
C3—C4—C5	121.2 (3)	C10—C11—H11A	118.3
C3—C4—H4A	119.4	C12—C11—H11A	118.3
C5—C4—H4A	119.4	C11—C12—N2	121.3 (3)
C6—C5—C4	121.2 (3)	C11—C12—H12A	119.4
C6—C5—H5A	119.4	N2—C12—H12A	119.4
C4—C5—H5A	119.4	C9—C13—N2	121.1 (3)
C1—C6—C5	116.5 (3)	C9—C13—C14	109.8 (3)
C1—C6—C7	121.8 (3)	N2—C13—C14	129.1 (2)
C5—C6—C7	121.7 (3)	O2—C14—N1	125.2 (3)
N1—C7—C6	112.7 (2)	O2—C14—C13	129.4 (3)
N1—C7—H7A	109.0	N1—C14—C13	105.4 (2)
C6—C7—H7A	109.0		
			
C6—C1—C2—C3	2.8 (6)	C13—C9—C10—C11	0.7 (4)
C1—C2—C3—C4	−1.9 (6)	C8—C9—C10—C11	−179.4 (2)
C2—C3—C4—C5	0.6 (6)	C9—C10—C11—C12	−0.1 (4)
C3—C4—C5—C6	−0.3 (6)	C10—C11—C12—N2	−1.1 (4)
C2—C1—C6—C5	−2.4 (5)	C13—N2—C12—C11	1.4 (4)
C2—C1—C6—C7	177.4 (3)	C10—C9—C13—N2	−0.2 (4)
C4—C5—C6—C1	1.1 (5)	C8—C9—C13—N2	179.9 (2)
C4—C5—C6—C7	−178.7 (3)	C10—C9—C13—C14	178.1 (2)
C14—N1—C7—C6	91.4 (3)	C8—C9—C13—C14	−1.8 (3)
C8—N1—C7—C6	−87.6 (3)	C12—N2—C13—C9	−0.8 (4)
C1—C6—C7—N1	−88.5 (4)	C12—N2—C13—C14	−178.8 (2)
C5—C6—C7—N1	91.3 (4)	C8—N1—C14—O2	177.7 (3)
C14—N1—C8—O1	−179.2 (3)	C7—N1—C14—O2	−1.4 (4)
C7—N1—C8—O1	−0.1 (4)	C8—N1—C14—C13	−1.1 (3)
C14—N1—C8—C9	0.0 (3)	C7—N1—C14—C13	179.8 (2)
C7—N1—C8—C9	179.2 (2)	C9—C13—C14—O2	−176.9 (3)
O1—C8—C9—C10	0.5 (5)	N2—C13—C14—O2	1.2 (5)
N1—C8—C9—C10	−178.7 (3)	C9—C13—C14—N1	1.8 (3)
O1—C8—C9—C13	−179.6 (3)	N2—C13—C14—N1	179.9 (2)
N1—C8—C9—C13	1.1 (3)		

### Hydrogen-bond geometry (Å, °)

**Table:**

*D*—H···*A*	*D*—H	H···*A*	*D*···*A*	*D*—H···*A*
C10—H10A···N2^i^	0.93	2.46	3.386 (3)	177

Symmetry codes: (i) −*x*, *y*−1/2, −*z*+1/2.
